# Inhibition of DEPDC1A, a Bad Prognostic Marker in Multiple Myeloma, Delays Growth and Induces Mature Plasma Cell Markers in Malignant Plasma Cells

**DOI:** 10.1371/journal.pone.0062752

**Published:** 2013-04-30

**Authors:** Alboukadel Kassambara, Matthieu Schoenhals, Jérôme Moreaux, Jean-Luc Veyrune, Thierry Rème, Hartmut Goldschmidt, Dirk Hose, Bernard Klein

**Affiliations:** 1 INSERM, U1040, Montpellier, France; 2 CHU Montpellier, Institute of Research in Biotherapy, Montpellier, France; 3 Medizinische Klinik V, Universitätsklinikum Heidelberg and Nationales Centrum für Tumorerkrankungen, Heidelberg, Germany; 4 Université MONTPELLIER1, UFR Médecine, Montpellier, France; Mayo Clinic, United States of America

## Abstract

High throughput DNA microarray has made it possible to outline genes whose expression in malignant plasma cells is associated with short overall survival of patients with Multiple Myeloma (MM). A further step is to elucidate the mechanisms encoded by these genes yielding to drug resistance and/or patients’ short survival. We focus here on the biological role of the DEP (for Disheveled, EGL-10, Pleckstrin) domain contained protein 1A (DEPDC1A), a poorly known protein encoded by *DEPDC1A* gene, whose high expression in malignant plasma cells is associated with short survival of patients. Using conditional lentiviral vector delivery of *DEPDC1A* shRNA, we report that *DEPDC1A* knockdown delayed the growth of human myeloma cell lines (HMCLs), with a block in G2 phase of the cell cycle, p53 phosphorylation and stabilization, and p21^Cip1^ accumulation. *DEPDC1A* knockdown also resulted in increased expression of mature plasma cell markers, including CXCR4, IL6-R and CD38. Thus DEPDC1A could contribute to the plasmablast features of MMCs found in some patients with adverse prognosis, blocking the differentiation of malignant plasma cells and promoting cell cycle.

## Introduction

Multiple myeloma (MM) is a heterogeneous clonal plasma-cell disorder in terms of molecular abnormalities, proliferation, and differentiation. Multiple myeloma cells (MMCs) from almost all patients harbor chromosomal abnormalities detected by iFISH [1] and at least 7 molecular groups have been identified in previously-untreated patients using high throughput gene expression profiling [2]. Numerous genes whose expressions in MMCs are associated with adverse or good prognosis have been identified and used to build gene expression-based prognostic scores [3], [4], [5], [6], [7], [8], [9]. Some of these genes encode for proteins involved in DNA replication, repair and recombination, as it is the case in other cancers [10], [11], [12], [13].

Whereas a majority of recent studies concur to indicate that the myeloma progenitor cell, able to form colonies in semi-solid culture medium vitro or tumors in animal models, express plasma cell markers (lack of CD20 et expression of CD138) [14], [15], [16], it is well recognized that MMCs in patients with poor prognosis are less differentiated than normal bone marrow plasma cells expressing plasmablast cytological markers, and secreting lower levels of Ig [17]. We report here that the DEPDC1A protein – for DEP (for Disheveled, EGL-10, Pleckstrin) domain contained protein 1A – could be involved in this undifferentiated stage of MMCs in some patients. The biological function of DEPDC1A is poorly known, with only 4 published reports showing it is a bad prognostic factor in patients with bladder, breast or lung cancers [18], [19], [20]. In addition, a knockdown of DEPDC1A inhibited growth of bladder cancer cell line [21].

We report here that *DEPDC1A* gene expression in MMCs of previously-untreated patients with MM is associated with adverse prognosis, and that *DEPDC1A* knockdown induces growth retardation and overexpression of genes coding for mature plasma cell markers in multiple myeloma cell lines.

## Results

### Increased Expression of *DEPDC1A* Gene in Multiple Myeloma Cells Compared to Normal Bone Marrow Plasma Cells in Association with a Poor Prognosis

**Figure 1:**
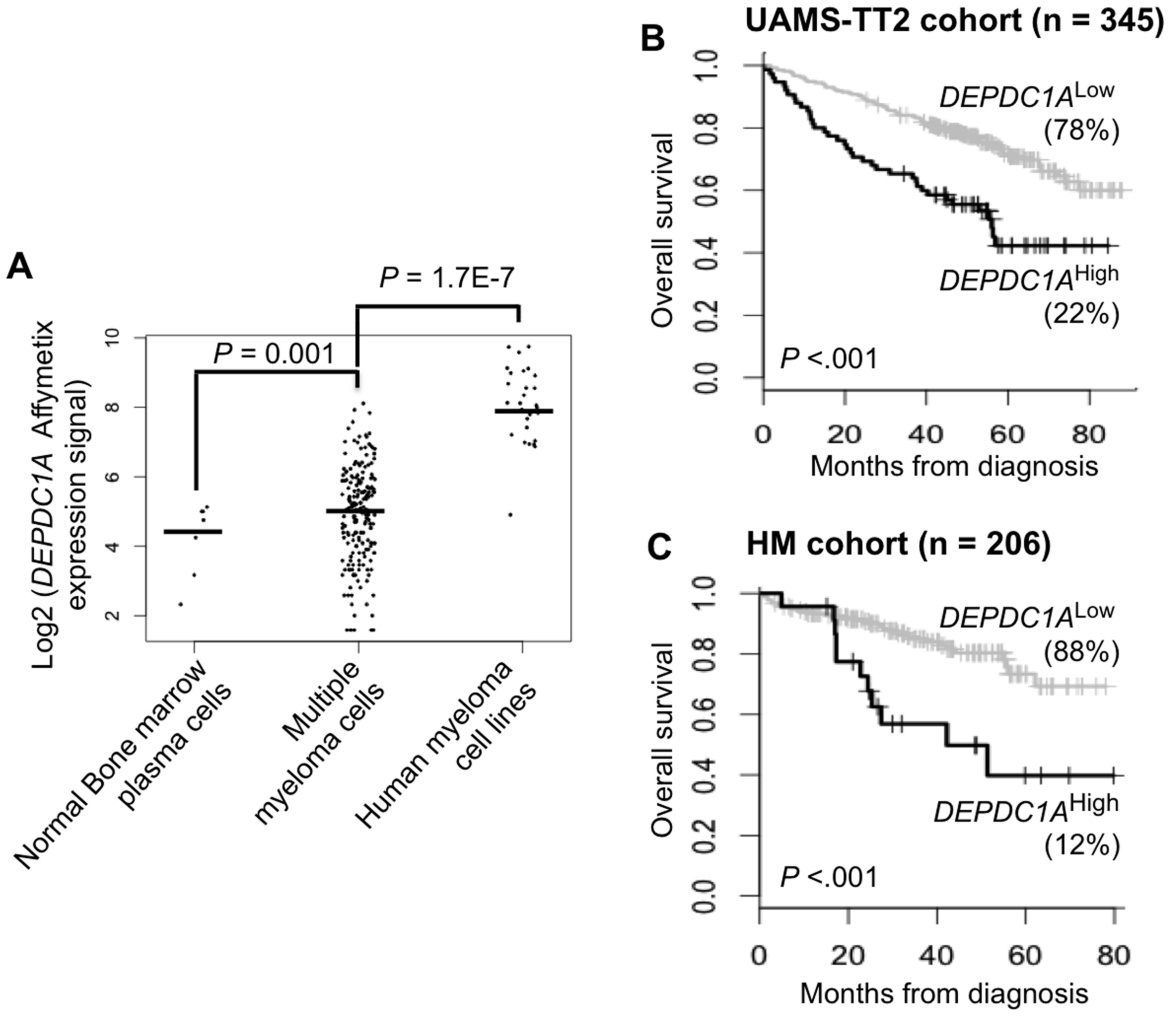
*DEPDC1A* gene is expressed in primary myeloma cells of patients with newly-diagnosed MM in association with a short overall survival. **A.** *DEPDC1A* gene expression was assayed using Affymetrix microarray in normal bone marrow plasma cells (BMPCs, n = 7), primary multiple myeloma cells (MMCs) of 206 newly-diagnosed patients with MM and 20 Human Myeloma Cell Lines (HMCLs). Data are the log2 MAS5-normalized expression signal of *DEPDC1A* probe set 222958_s_at (the *DEPDC1A* probe set yielding the highest variance) in the different cell populations and statistical comparison was done with a Mann Whitney test. **B.** Prognostic value of *DEPDC1A* expression using UAMS-TT2 cohort of patients. **C.** Prognostic value of *DEPDC1A* expression using HM cohort of patients. The R Maxstat function was used to compute the cutoff yielding to the maximum difference in overall survival between patients with low or high *DEPDC1A* expression. Kaplan Meir survival curves of patients with high (black line) or low (gray line) *DEPDC1A* expression signal are shown.

*DEPDC1A* gene expression was significantly increased (*P* = .001) in MMCs of previously-untreated patients compared to normal BMPCs and in HMCLs compared to primary MMCs (Figure 1A). Affymetrix data (probe set 222958_s_at) were validated by real time RT-PCR (*P* = .005, Supplementary Figure S1A). A high *DEPDC1A* expression could predict for shorter overall survival in 2 independent large cohorts of previously-untreated patients. Using Maxstat R function, 22% of the patients of UAMS-TT2 cohort with the highest *DEPDC1A* expression had an overall survival of 56 months versus not reached in the remaining patients (Figure 1B, *P*<.001). Twelve % of the patients of the HM cohort with high *DEPDC1A* expression had an overall survival of 42.2 months versus not reached for the remaining patients (Figure 1C, *P*<.001). MMCs of previously-untreated patients can be classified in 8 molecular groups [2] and *DEPDC1A* expression was significantly increased in the proliferation (PR) group and decreased in the low bone disease (LB), hyperdiploidy (HY), and myeloid (MY) groups (*P* ≤.05, Supplementary Figure S1B).

As DEPDC1A could be involved in cell proliferation [21], the prognostic value of *DEPDC1A* gene expression was compared to that of a gene expression based proliferation index (GPI) recently designed by our group [5] or of the Proliferation (PR) stratification designed by UAMS [2]. In univariate Cox analysis, the 3 parameters were significant. When compared two by two, *DEPDC1A* remained an independent prognosis factor when tested with PR stratification, and only *DEPDC1A* remained significant when tested with GPI. When the 3 parameters were tested together, only *DEPDC1A* remained significant (Supplementary Table S2).

### Knockdown of *DEPDC1A* Gene Delays the Cell Growth of Human Myeloma Cell Lines (HMCLS)

**Figure 2:**
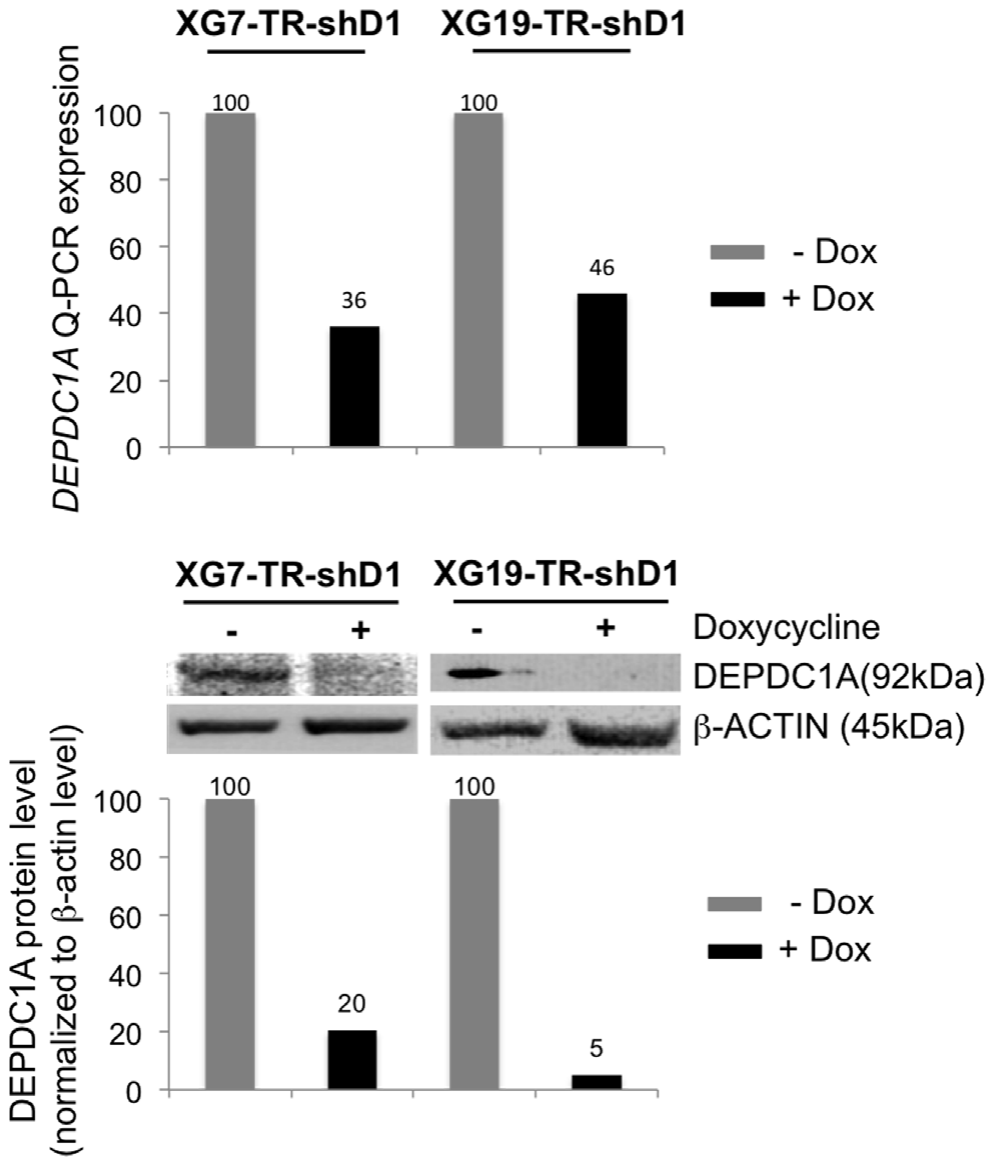
Knockdown of *DEPDC1A* expression using shRNA. XG7-TR-shD1 and XG19-TR-shD1 cells were treated for 6 days with doxycycline (dox). Dox-induced knockdown of *DEPDC1A* expression was assayed using real time PCR and western blotting. Results are those of one experiment representative of three. Western lots were quantified by densitometry using NIH ImageJ software (National Institutes of Health, Bethesda, MD, USA) and levels of DEPDC1A protein normalized according to those of β-actin, and giving the arbitrary value of 100 in cells not treated with dox.

**Figure 3:**
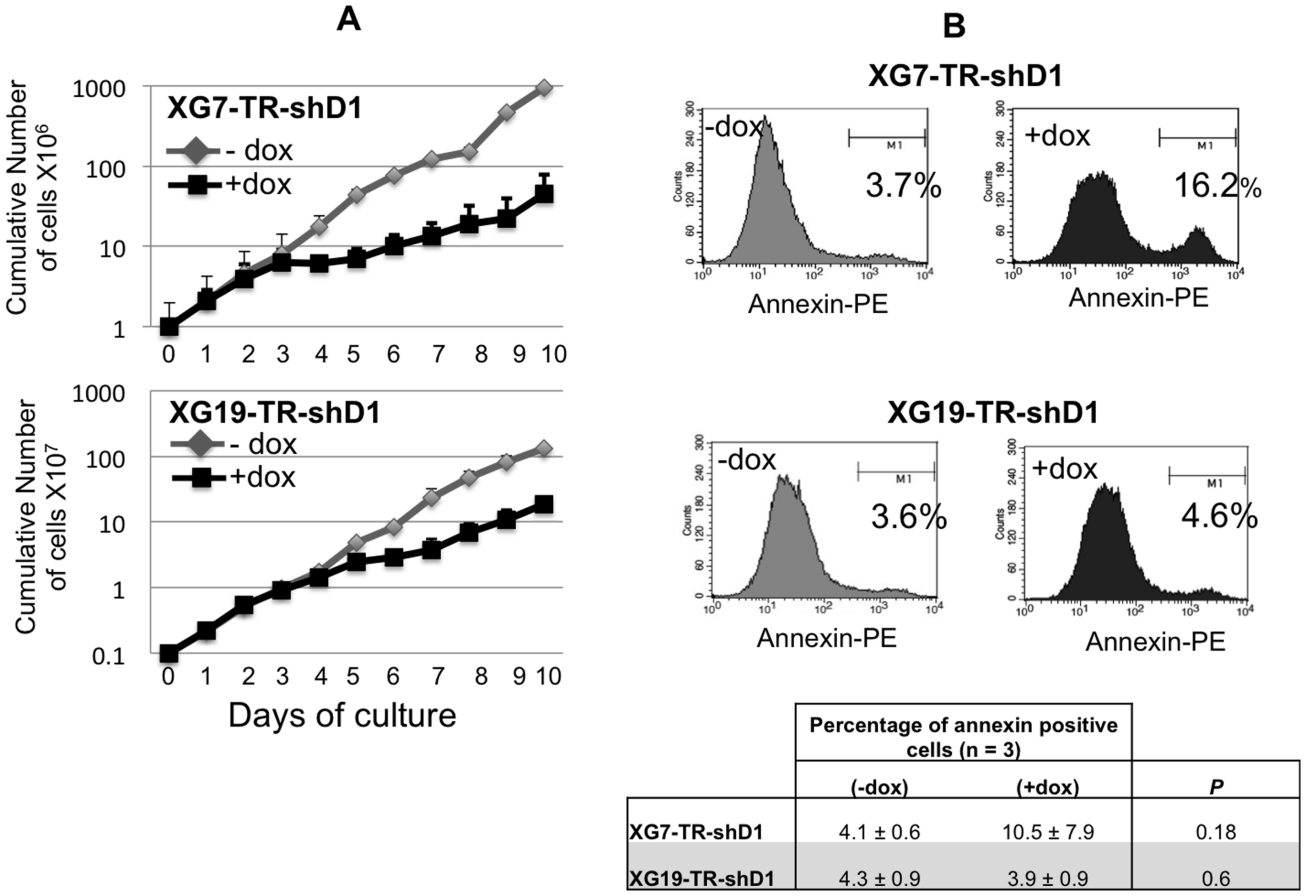
Knockdown of *DEPDC1A* expression delays myeloma cell line growth. The HMCL-TR-shD1 were cultured for 10 days with or without doxycycline (dox). Data are the mean counts ± SD of viable cells using trypan blue exclusion of 3 separate experiments. Given the log scale representation, standard errors bars could be too small to be visible. **B.** The HMCL-TR-shD1 were cultured for 6 days with or without dox and annexin V^+^ apoptotic cells evaluated by flow cytometry. Data are FACS data of one representative experiment and mean values ± SD of 3 separate experiments. Statistical analysis was done with a paired t-test.

**Figure 4:**
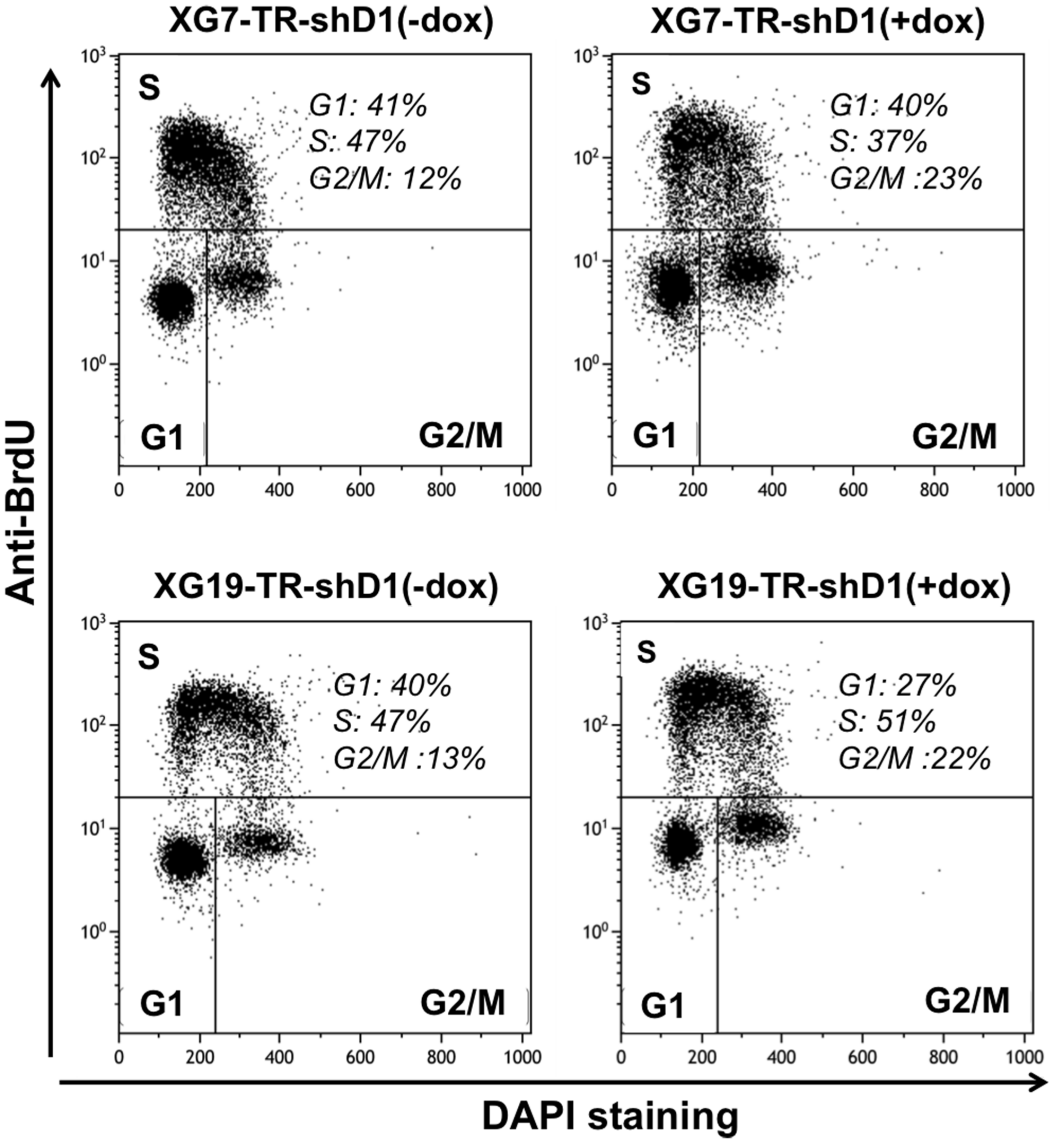
Knockdown of *DEPDC1A* expression blocks cell cycle in myeloma cell lines. XG7-TR-shD1 and XG19-TR-shD1 cells were cultured with or without doxycycline (dox) for 6 days and cell cycle was quantified using BrdU incorporation and labelling with an anti-BrdU antibody and DAPI. Data are those of one experiment representative of three.

Two wild-type *TP53* HMCLs (XG7 and XG19) were first transduced with a lentivirus containing the tetracycline repressor (TR) that repressed the Tet operator (HMCL-TR) and HMCL-TRs were then transduced with a lentiviral vector containing a *DEPDC1A* shRNA (shD1) linked to the *GFP* gene under the control of the Tet operator (HMCL-TR-shD1). As expected, HMCL-TR-shD1 did not express GFP and adding doxycycline (dox) induced GFP expression (Supplementary Figure S2). Doxycycline reduced *DEPDC1A* RNA by 50–60% (*P*<.001) and DEPDC1A protein by 80–95% (*P*<.001) in the 2 HMCL-TR-shD1 (Figure 2). An efficient inhibition of *DEPDC1A* expression by the shRNA was only observed after 3–5 days culture as shown in supplementary Figure S3. Doxycycline had no effect on HMCL-TR, which did not contain *DEPDC1A* shRNA (data not shown). Adding doxycycline induced growth delay of the 2 HMCL-TR-shD1, which becomes visible 4–5 days after adding doxycycline (Figure 3A). It had no effect on control HMCL-TR (data not shown). In 3 separate experiments, the doubling time of XG7-HMCL-TR-shD1 cells was significantly (*P*<.05) increased 2 fold (from 1 day to 2 days) by adding doxycycline, and that of XG19-HMCL-TR-shD1 1.7 fold (from 0.9 day to 1.6 days, *P*<.05). The growth retardation induced by *DEPDC1A* knockdown was not due to a significant induction of apoptosis (Figure 3B), but to a partial blockade of cell cycle in the G2/M phase (Figure 4). Using BrdU incorporation and labelling with DAPI dye and an anti-BrdU antibody, the percentage of cells in the G2/M phase was increased 1.9 fold and 1.7 fold respectively in XG7-HMCL-TR-shD1 and XG19-HMCL-TR-shD1 in 3 separate experiments (P<.05, Table 1). FACS data of a representative experiment are displayed on Figure 4. The MFI of the labelling with anti-BrdU antibody was similar for cells treated or not with doxycycline, suggesting no delay in the DNA synthesis rate (Figure 4). To exclude off-targets of *DEPDC1A* shRNA, we used a siRNA targeting the non-coding 3′ part of *DEPDC1A* mRNA. This *DEPDC1A* siRNA had the same biological effect as the *shDEPDC1A*, which targets *DEPDC1A* coding sequence: reduction of *DEPDC1A* mRNA by 47%, delay the growth of XG7 cells (Supplementary Figure S4), with partial accumulation of cells in the G2 phase (supplementary Figure S4). Of interest, overexpressing *DEPDC1A* gene lacking the non-coding 3′ sequence targeted by the *DEPDC1A* siRNA abrogated the siRNA-mediated growth inhibitory effect and delay in G2 phase as shown in Supplementary Figure S4.

**Table 1:** Knockdown of *DEPDC1A* expression induces a partial blockade in the G2/M phase.

	XG7-TR-shD1		XG19-TR-shD1	
	(−dox)	(+dox)	(−dox)	(+dox)
**G1**	37.2±5	37.5±3.7	42.6±3.2	29.4±3.2*
**S**	50.6±5.2	41.9±7.4*	48.8±2.6	53.2±2.6
**G2/M**	12.2±0.3	20.5±3.5*	8.5±5.9	17.3±5.8*

XG7-TR-shD1 or XG19-TR-shD1 cells were cultured with or without dox for 6 days and cell cycle was quantified using BrdU incorporation and labelling with an anti-BrdU antibody and DAPI. Data are the mean percentages ± SD of cells in each phase of the cell cycle (G1, S, G2/M) of three separate experiments.

* indicates the mean value in the dox group is significantly different than that in the control group using a paired t test (*P*<.05).

**Figure 5:**
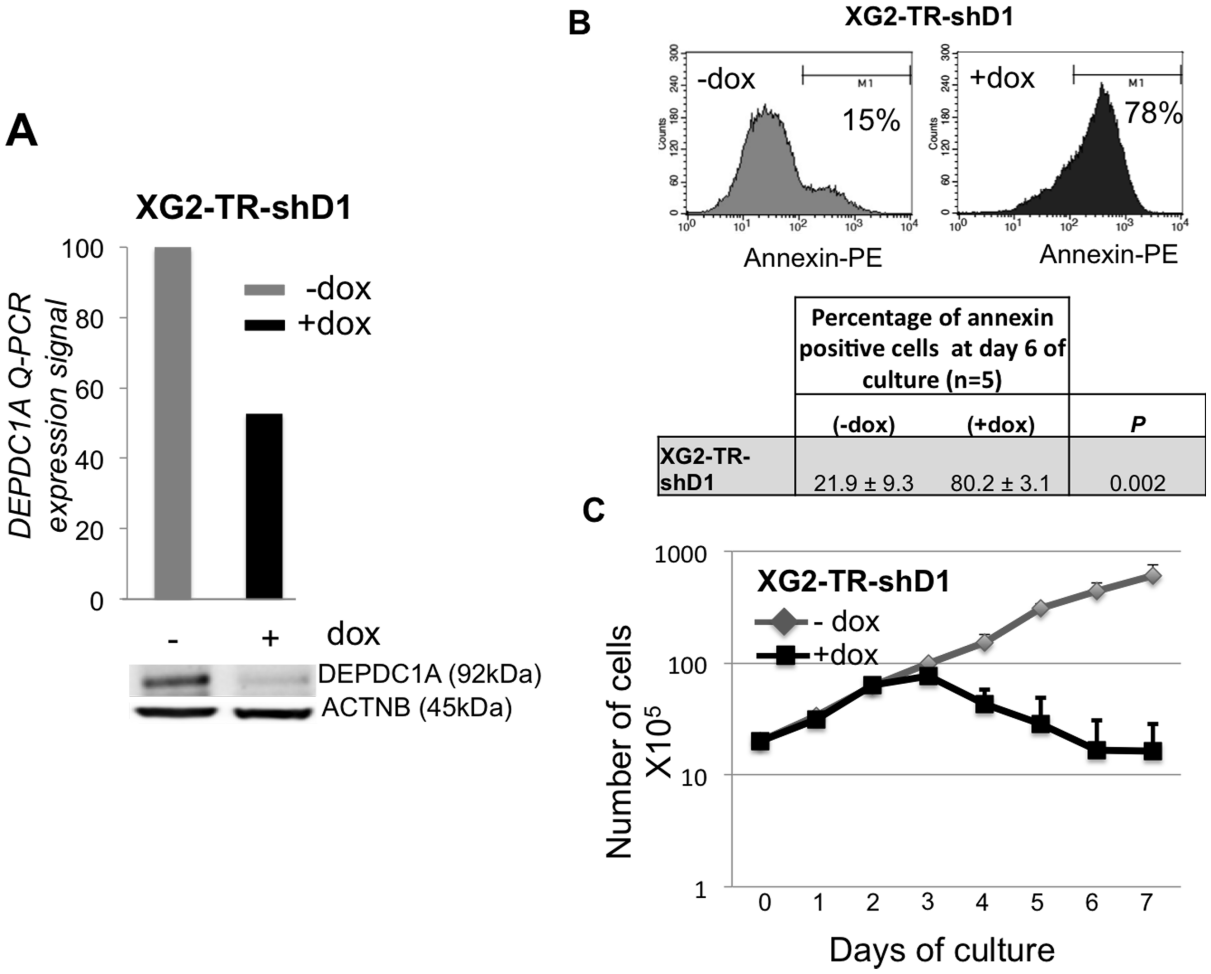
Knockdown of *DEPDC1A* induced apoptosis in *TP53*-mutated cell line. **A.** XG2-TR-shD1 cells were treated for 3 days with doxycycline (dox). Doxycycline-induced knockdown of *DEPDC1A* gene or protein expression were assayed using real time PCR or western blotting. **B.** XG2-TR-shD1 cells were cultured for 6 days with or without dox and annexin V^+^ apoptotic cells evaluated by flow cytometry. Data are FACS data of one representative experiment and mean values ± SD of 3 separate experiments. Statistical analysis was done with a paired t-test. **C.** XG2-TR-shD1 cells were cultured for 7 days with or without doxycycline (dox). Data are the mean counts ± SD of viable cells using trypan blue exclusion of 3 separate experiments. Given the log scale representation, standard errors bars could be too small to be visible.

We next evaluated the role of DEPDC1A in a dox inducible *TP53-*mutated cell line (XG2-TR), which was transduced with the *DEPDC1A* shRNA lentiviral vector. Adding doxycycline reduced DEPDC1A RNA by 50% (*P*<.001) and protein by 90% (*P*<.001) (Figure 5A). DEPDC1A knockdown induced a dramatic apoptosis for XG2-HMCL-TR-shD1 cells, (80.2%±3.1%, *P* = .002) and a stop in cell growth after 3 days of culture (Figures 5B and 5C). Due to the massive apoptosis in XG2-HMCL-TR-shD1 cells, the effect of DEPDC1A knockdown on cell cycle could not be investigated in XG2 cells.

### Regulation of Signaling Pathway by *DEPDC1A* Knockdown

**Figure 6:**
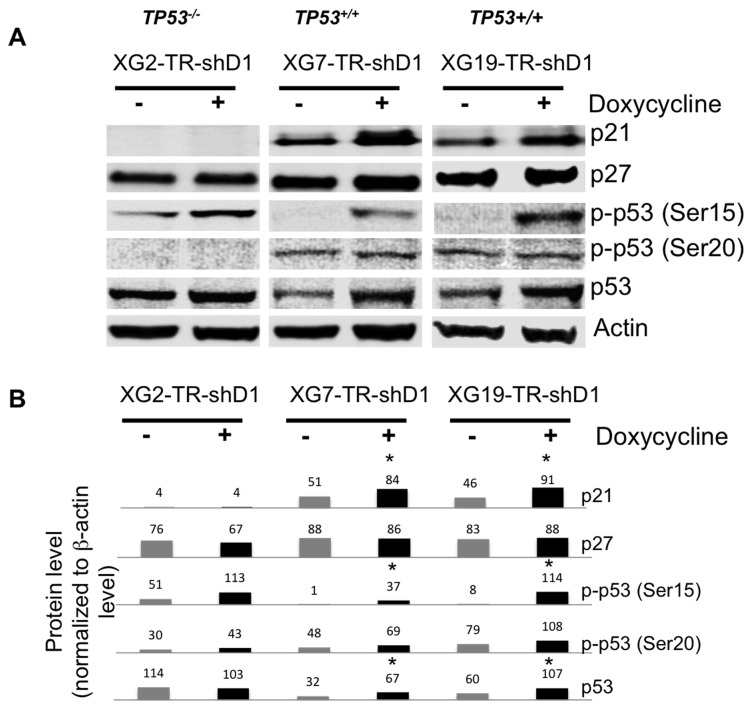
Knockdown of *DEPDC1A* induced p53 phosphorylation and stabilization, and p21^Cip1^ accumulation. **A.** HMCL-TR-shD1 cells were cultured for 4-6 days with or without dox and protein detection was assayed using western blot analysis. Membranes were stained with rabbit anti-p27^Kip1^, anti-p53, anti-Phospho-p53 (Ser15 or Ser20) and mouse anti-p21^Cip1^ antibodies. A mouse monoclonal anti-β-actin antibody was used as control. The antibody binding was revealed using peroxidase-conjugated secondary antibodies and an enhanced chemiluminescence detection system. **B.** Western lots were quantified by densitometry using NIH ImageJ software (National Institutes of Health, Bethesda, MD, USA) and protein levels normalized according to those of β-actin. Data are the mean normalized protein expressions of 3 experiments. *indicates a significant change (*P*<.05) in dox-treated cells compared to untreated ones using a Wilcoxon test.

Given the cell cycle delay induced by *DEPDC1A* knockdown, the expression of some major proteins regulating cell cycle was investigated. *DEPDC1A* knockdown resulted in p21^Cip1^ induction in association with p53 phosphorylation on Ser-15, leading to p53 stabilization (Figure 6). P27^Kip1^ expression was not affected (Figure 6). In XG2, the HMCL carrying *TP53* mutated genes, a high level of mutant p53 could be detected and *DEPDC1A* knockdown induced no induction of p21^cip1^ and no change in p27^Kip1^ level (Figure 6).

### *DEPDC1A* Knockdown Increases Expression of Markers of Mature Plasma Cells

Genes whose expression was regulated by *DEPDC1A* knockdown were identified using Affymetrix U133 plus 2.0 microarrays performing 3 independent experiments for each of the 3 HMCLs. Wild-type *TP53* XG7 and XG19 cells were treated for 5 days with or without dox and mutated *TP53* XG2 cells for 3 days before apoptosis occurrence. Statistical analysis was done with Affymetrix GCOS (GeneChip Operating Software) software and the *P*-value are adjusted with Benjamini-Hochberg multiple testing correction. Crossing the gene lists obtained in the 3 independent experiments for each HMCL, 34 genes/ESTs were upregulated (fold change ≥1.5, *P≤*.05) and 125 downregulated (fold change ≤0.67, *P≤*.05) by *DEPDC1A* knockdown in XG7 HMCL (supplementary Table S3), 56 genes/ESTs upregulated (fold change ≥1.5, *P≤*.05) and 87 downregulated (fold change ≤0.67, *P≤*.05) in XG19 HMCL (supplementary Table S4). Twelve genes/ESTs were commonly upregulated (fold change ≥1.5, *P≤*.05) and 39 downregulated (fold change ≤0.67, *P≤*.05) in XG7 and XG19 HMCLs (Table 2). Regarding XG2 cells, 52 genes/ESTs were upregulated (fold change ≥1.5, *P≤*.05) and 60 downregulated (fold change ≤0.67, *P≤*.05) by *DEPDC1A* knockdown in XG2 HMCL (supplementary Table S5), and only 13 genes were commonly deregulated in XG7 and XG19 cells in one hand and XG2 cells in the other hand (Table 3), likely due to the dramatic apoptotic program induced by *shDEPDC1A* in XG2 cells.

**Table 2:** Common genes deregulated by *DEPDC1A* knockdown in XG7 and XG19 human myeloma cell lines carrying wild-type TP53 genes.

Genes significantly upregulated by DEPDC1A knockdown			Genes significantly downregulated by DEPDC1A knockdown		
Probe sets	Gene Name	Fold Change	Probe sets	Gene Name	Fold Change
1555340_x_at	*RAP1A*	11.1	228636_at	*BHLHE22*	0.23
216001_at	*PRAMEF12*	2.7	212353_at	*SULF1*	0.25
224724_at	*SULF2*	2.5	228499_at	*PFKFB4*	0.42
217028_at	*CXCR4*	2.3	201627_s_at	*INSIG1*	0.42
1553970_s_at	*CEL*	2.1	225429_at	*PPP6C*	0.43
205692_s_at	*CD38*	1.8	214930_at	*SLITRK5*	0.44
228003_at	*RAB30*	1.7	227728_at	*PPM1A*	0.46
203409_at	*DDB2*	1.7	221844_x_at	*SPCS3*	0.49
207813_s_at	*FDXR*	1.7	200748_s_at	*FTH1*	0.50
218953_s_at	*PCYOX1L*	1.6	235545_at	*DEPDC1*	0.50
201362_at	*IVNS1ABP*	1.5	219274_at	*TSPAN12*	0.52
205945_at	*IL6R*	1.5	201170_s_at	*BHLHE40*	0.53
			224680_at	*TMED4*	0.53
			212690_at	*DDHD2*	0.54
			223249_at	*CLDN12*	0.55
			203971_at	*SLC31A1*	0.55
			224958_at	*NUFIP2*	0.56
			203637_s_at	*MID1*	0.56
			219410_at	*TMEM45A*	0.56
			234994_at	*TMEM200A*	0.58
			212192_at	*KCTD12*	0.58
			203164_at	*SLC33A1*	0.59
			212635_at	*TNPO1*	0.60
			203216_s_at	*MYO6*	0.61
			202887_s_at	*DDIT4*	0.61
			212371_at	*PPPDE1*	0.61
			230619_at	*ARNT*	0.61
			219221_at	*ZBTB38*	0.62
			225112_at	*ABI2*	0.63
			209425_at	*AMACR*	0.63
			201649_at	*UBE2L6*	0.64
			226689_at	*CISD2*	0.64
			202068_s_at	*LDLR*	0.65
			238065_at	*TPM3*	0.65
			205830_at	*CLGN*	0.65
			202245_at	*LSS*	0.65
			212482_at	*RMND5A*	0.66
			36711_at	*MAFF*	0.66
			224445_s_at	*ZFYVE21*	0.67

XG7-TR-shD1 and XG19-TR-shD1 cells were cultured with or without doxycycline for 5 days. Genes whose expression is deregulated by *DEPDC1A* knockdown were identified using Affymetrix U133 plus 2.0 microarrays. Data are the common genes significantly (P≤.05) upregulated (≥1.5 fold) or downregulated (≤0.67 fold) by *DEPDC1A* knockdown in the 2 human multiple myeloma cell lines.

**Table 3:** Common genes deregulated by *DEPDC1A* knockdown in the 2 wild-type *TP53* XG19 and XG7 human myeloma cell lines and in the mutated *TP53* XG2 human myeloma cell line.

Probe sets	Gene Name	Fold Change	Category
1555340_x_at	RAP1A	8.49	**Genes significantly upregulated by DEPDC1A knockdown**
227728_at	PPM1A	0.44	**Genes significantly downregulated by DEPDC1A knockdown**
218817_at	SPCS3	0.46	
235545_at	DEPDC1	0.47	
221844_x_at	SPCS3	0.55	
225429_at	PPP6C	0.55	
224680_at	TMED4	0.55	
212690_at	DDHD2	0.57	
238065_at	TPM3	0.61	
225112_at	ABI2	0.62	
224445_s_at	ZFYVE21	0.62	
226689_at	CISD2	0.64	
202666_s_at	ACTL6A	0.65	

XG2-TR-shD1 cells were cultured with or without doxycycline for 3 days and XG7-TR-shD1 and XG19-TR-shD1 cells for 5 days. Genes whose expression is deregulated by *DEPDC1A* knockdown were identified using Affymetrix U133 plus 2.0 microarrays. Data are the common gene significantly significantly (P≤.05) upregulated (≥1.5 fold) or downregulated (≤0.67 fold) by *DEPDC1A* knockdown in the 3 human multiple myeloma cell lines.

**Figure 7:**
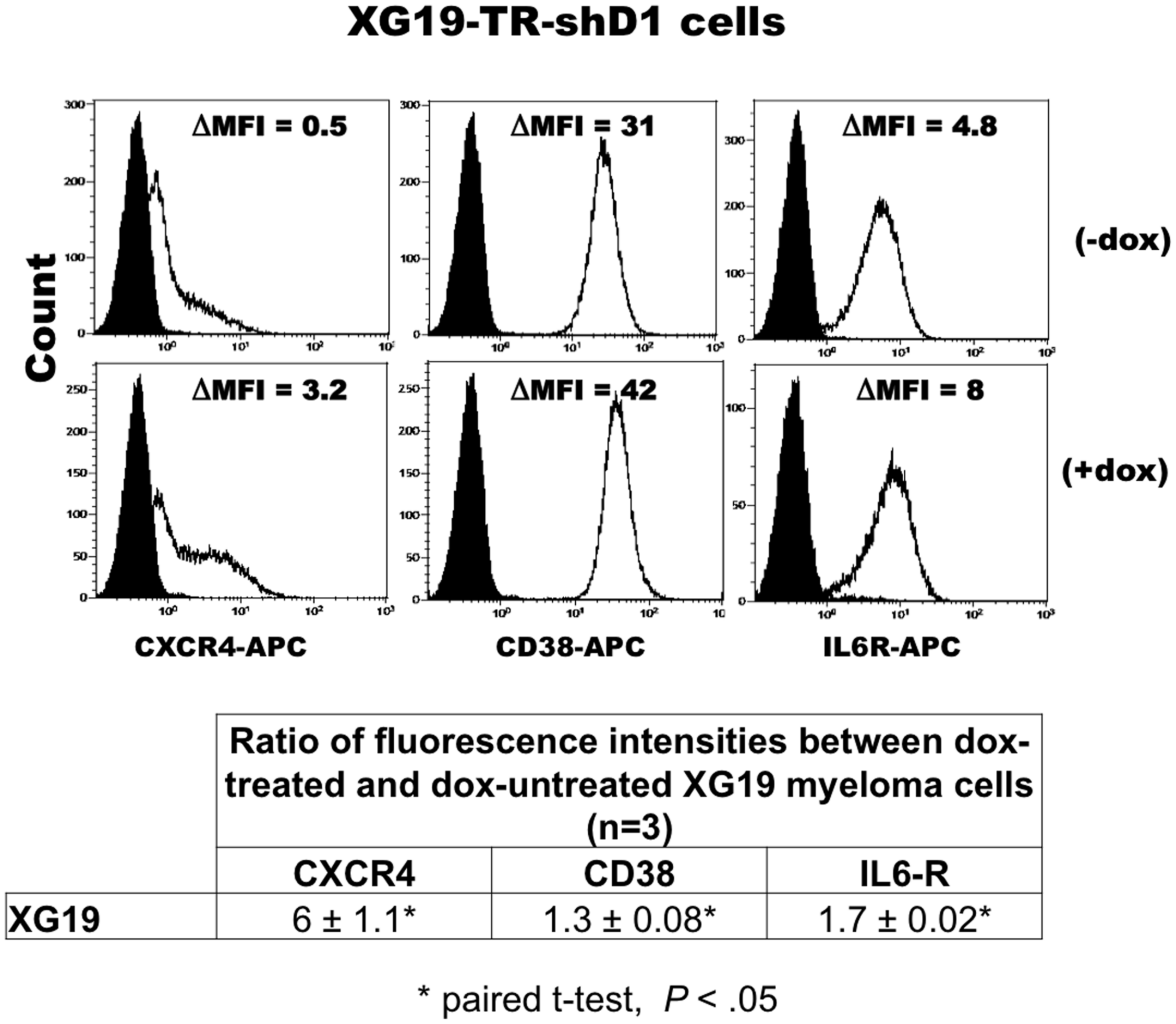
*DEPDC1A* knockdown increases expression of markers of mature plasma cells in myeloma cell lines. XG19-TR-shD1 cells were cultured with or without doxycycline (dox) for 6 days and cells were stained with phycoerythrin-conjugated anti-CXCR4 mAb, anti-IL6R mAb, anti-CD38 mAb (continuous line) or isotype-matched control mAbs (black full histogram). Facs data are those of one experiment representative of three. ΔMFI is the difference between the mean fluorescence intensity (MFI) with the antigen-specific antibody and MFI with isotype-matched control mAb. The table indicates the mean ratio of ΔMFI between dox-treated and dox-untreated XG19 myeloma cells in 3 independent experiments. *indicates a significant increase in CXCR4, IL6R or CD38 expression using a paired t-test (*P*<.05).

The majority of genes upregulated in HMCL-TR-shD1 upon *DEPDC1A* knockdown encode for proteins, we and others have reported to be markers of normal mature plasma cells including *CD38* and its receptor *PECAM1/CD31, CXCR4, IL6R, CD9, SULF2, FGFR2, HES1* and *HES2* [*22]*, [23**]. We checked that *DEPDC1A* knockdown could significantly (*P*<.05) upregulate the expression of CXCR4, IL-6R or CD38 proteins in XG19 cells (Figure 7) or XG7 cells (supplementary Figure S5) using flow cytometry in 3 independent experiments.

### *DEPDC1A^High^* MMCs have a Plasmablastic Signature and *DEPDC1A^Low^* MMCs a Mature Plasma Cell One

**Figure 8:**
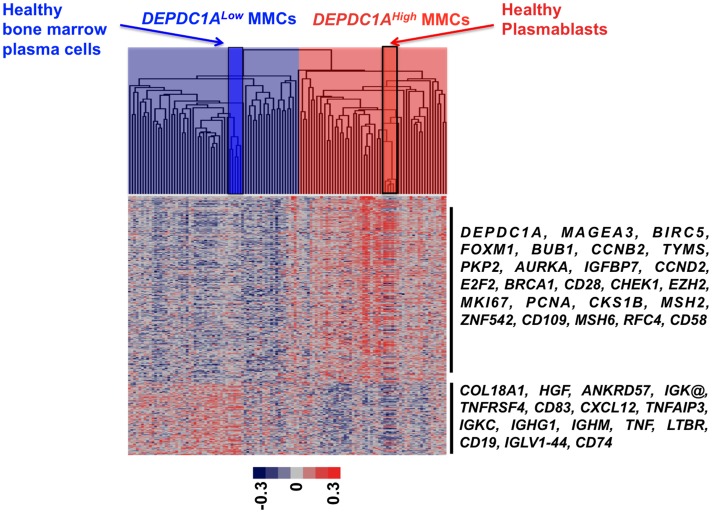
*DEPDC1A* gene signature cluster *DEPDC1A* ^High^ MMCs with healthy plasmablasts and *DEPDC1A* ^Low^ MMCs with mature bone marrow plasma cells. Using the 320 genes differentially expressed between *DEPDC1A* ^High^ and *DEPDC1A* ^Low^ MMCs, *DEPDC1A* ^High^ MMCs (light red) of previously-untreated patients cluster together with healthy plasmablasts (red) and *DEPDC1A* ^Low^ MMCs (light blue) cluster with mature healthy bone marrow plasma cells. The color of each cell in the tabular image represents the expression level of each gene (red, expression higher than the mean; blue, expression lower than the mean; increasing color intensity represents a higher magnitude of deviation from the mean). Arrows highlight some remarkable genes.

We looked for differences in gene expression profiles between MMCs of 25% of patients with the highest *DEPDC1A* expression (*DEPDC1A^High^*) and those of 25% of patients with the lowest *DEPDC1A* expression (*DEPDC1A^Low^*). 320 genes/ESTs were differentially expressed between D*EPDC1A^Low^* and *DEPDC1A^High^* MMCs using SAM supervised analysis (1000 permutation, fold change ≥2, supplementary Table S6). Using Gene Ontology, *DEPDC1A^High^* MMC genes are enriched in genes coding for proteins involved in cell cycle or DNA replication (Supplementary Table S6). Conversely, *DEPDC1A^Low^* MMC genes are enriched in genes coding for intercellular communication signals, immunoglobulin heavy chains, and signal transduction (Supplementary Table S6). Using unsupervised clustering with these 320 DEPDC1A genes, *DEPDC1A^High^* MMCs clustered together with healthy plasmablasts (Spearman correlation coefficient, 0.6; *P* = .001) and *DEPDC1A^Low^* MMCs with healthy bone marrow plasma cells (Spearman correlation coefficient, 0.4; *P* = .001; Figure 8). Altogether, these data indicate *DEPDC1A^Low^* MMCs display a mature plasma cell signature, and *DEPDC1A^High^* MMCs a plasmablast one.

## Discussion

We report that a high *DEPDC1A* gene expression in MMCs is associated with poor prognosis in two independent large series of previously-untreated patients with MM, and that a conditional knockdown of *DEPDC1A* affects the growth of multiple myeloma cell lines and induces markers of mature plasma cell. Regarding myeloma cell lines, two different biological effects were observed: a growth delay without major apoptosis in XG7 and XG19 cells carrying wild-type *TP53* genes and a dramatic apoptosis in the XG2 cells carrying mutated *TP53* genes. In wild-type *TP53* cell lines, the growth delay is explained by a partial blockade of myeloma cells in the G2/M phase of the cell cycle, in association with p53 phosphorylation and stabilization, p21^Cip1^ expression, and no change in p27^KIP1^. P21^Cip1^ is a cell cycle inhibitor acting on the G1/S phase, late S phase and G2/M phase checkpoints [24]. p53 phosphorylation could be induced by ATM and CHK2 kinases controlling the DNA Damage Response (DDR) [25]. But we failed to find *DEPDC1A* knockdown increases the phosphorylation of ATM and CHK2 or of ATR and CHK1 kinases (results not shown). Alternative hypotheses are that *DEPDC1A* knockdown could activate other kinases able to phosphorylate p53, such as DNA-dependent protein kinase [26]. Of note, DEPDC1A was recently shown to interact with zinc finger transcription factor ZNF224, a known transcriptional repressor [19]. The transcriptional repression by ZNF224 involves DNA epigenetic modifications, in particular binding and recruitment of KAP1 to DNA [27]. KAP1 is a scaffold protein activating the epigenetic machinery and participating in the DNA damage response [28]. Thus, we may speculate that DEPDC1A knockdown will release ZNF224, which could efficiently recruit KAP1 yielding to increased DNA damage response, activation of cell cycle checkpoints and delay in cell cycle. The high apoptosis induced by *DEPDC1A* knockdown in XG2 cells carrying mutated *TP53* genes could be understandable in light of data with wild-type *TP53* HMCLs. In these cells, there was no induction of p21^Cip1^ due to lack of wild-type p53. This could result in a lack of mitotic delay to check for DNA integrity and eventually repair of DNA defects, yielding to mitotic catastrophe [29].

A second current finding is that *DEPDC1A* knockdown induces markers of mature plasma cells in *TP53* wild-type HMCLs, including CD38, CXCR4, IL-6R. Plasma cell differentiation is mainly driven by the BLIMP1 inhibitory transcription factor [30]. BLIMP1 interacts with PRTM5, an arginine-specific histone methyltransferase, which in turn mediates symmetrical dimethylation of arginine 3 on histone H2A and/or H4 tails [31]. As PRTM5 is also a component of ZNF224 transcription complex [32], DEPDC1A could modulate PRTM5 activity and thus BLIMP1 activity, blocking full plasma cell differentiation. This function should be relevant for normal plasma cells as *DEPDC1A* is highly expressed in normal plasmablasts and downregulated upon their differentiation into plasma cells (see http://amazonia.transcriptome.eu/expression.php?probeId=222958_s_at&zone=PlasmaCell) [23], [33].

This function found using myeloma cell lines likely occurs in primary myeloma cells, since *DEPDC1A* is increased in myeloma cells of patients belonging to the proliferation molecular group. The fact that DEPDC1A promotes cell cycle and blocks differentiation of myeloma cells explains its value as an indicator of poor prognosis in multiple myeloma since both cell cycling and plasmablast phenotype of myeloma cells are associated with poor prognosis [34], [35]. Expression of *DEPDC1A* in human tissues can be visualized using our Amazonia! Web site (http://amazonia.transcriptome.eu/expression.php?probeId=222958_s_at). *DEPDC1A* is poorly expressed in the majority of tissues, except in human bone marrow and lymphoid organs. It is highly expressed in human embryonic pluripotent stem cells in agreement with its supposed ability to promote cell cycle and block differentiation. The current results together with previous data reporting a role of *DEPDC1A* in promoting epithelial cancer proliferation suggest that *DEPDC1A* could be an interesting target in multiple myeloma, with potentially limited toxicity.

## Materials and Methods

The study was approved by the ethics boards of the University Hospitals of Heidelberg and Montpellier. Multiple myeloma cells (MMCs) were purified from a series of 206 newly diagnosed patients with myeloma after written informed consent was given according to the Declaration of Helsinki.

### Patients’ Samples and Cell Lines

The XG1, XG2, XG3, XG4, XG5, XG6, XG7, XG10, XG11, XG12, XG13, XG16, XG19, and XG20 human myeloma cell lines (HMCLs) were obtained as described [36], [37], [38] and SKMM, OPM2, LP1 and RPMI8226 HMCLs purchased from ATTC (LGC Standards, France). These HMCLs were recently molecularly and phenotypically characterized [9]. MMCs of 206 patients with previously untreated MM were included in this study after written informed consent was given at the University hospitals of Heidelberg (Germany) or Montpellier (France) and agreement by the ethics boards of Heidelberg University and Montpellier University. These 206 patients were treated with high dose therapy (HDC) and autologous stem cell transplantation (ASCT) and this cohort is termed in the following Heidelberg-Montpellier (HM) cohort. Patients’ characteristics are available in Supplementary Table S1. Gene expression profiling (GEP) of purified MMCs was assayed using Affymetrix U133 2.0 plus microarrays as described [39] and data were normalized using the MAS5 Affymetrix algorithm with a scaling factor of 100. The.CEL and MAS5 files are deposited in the ArrayExpress public database (http://www.ebi.ac.uk/arrayexpress/), under accession number E-MTAB-362. Interphase-FISH-analysis was performed according to our previously reported protocol [40]. We also used publicly available Affymetrix data (http://www.ncbi.nlm.nih.gov/geo, GSE2658) of a cohort of 345 purified MMCs from previously untreated patients from the University of Arkansas for Medical Sciences (UAMS, Little Rock, AR). These patients were treated with total therapy 2 [41] and this cohort is termed in the following UAMS-TT2 cohort. Normal Healthy plasmablasts and healthy bone marrow plasma cells (BMPCs) were obtained as described [23], [42] and GEP data are available on ArrayExpress public database (http://www.ebi.ac.uk/arrayexpress/), under accession numbers E-MEXP-2360, E-MEXP-3034 and E-MEXP-2360.

### Real-time RT-PCR

RNA was converted to cDNA using the Qiagen’s QuantiTect Rev. Transcription Kit (Qiagen, Hilden, Germany). The assays-on-demand primers and probes and the TaqMan Universal Master Mix were used according to the manufacturer’s instructions (Applied Biosystems, Courtaboeuf, France). The measurement of gene expression was performed using the Roche LC480 Sequence Detection System. For each primer, serial dilutions of a standard cDNA were amplified to create a standard curve, and values of unknown samples were estimated relative to this standard curve in order to assess PCR efficiency. Ct values were obtained for *GAPDH* and the respective genes of interest during log phase of the cycle. Gene expression was normalized to that of *GAPDH* (ΔCt = Ct gene of interest – Ct GAPDH) and compared with the values obtained for a known positive control using the following formula: 100/2^ΔΔCt^ where ΔΔCt = ΔCt unknown –ΔCt positive control.

### Western Blot Analysis

Cells were lysed in 10mM Tris-HCl (pH 7.05), 50 mM NaCl, 50 mM NaF, 30 mM sodium pyrophosphate, 1% Triton X-100, 5 µM ZnCl_2_, 100 µM Na_3_VO4, 1 mM dithiothreitol, 20 mM β-glycerophosphate, 20 mM p-nitrophenol phosphate, 20 µg/ml aprotinin, 2.5 µg/ml leupeptin, 0.5 mM phenylmethylsulfonyl fluoride, 0.5 mM benzamidine, 5 µg/ml pepstatin and 50 nM okadaic acid. Lysates were resolved on 12% sodium dodecyl sulfate-polyacrylamide gel electrophoresis and transferred to a nitrocellulose membrane (Schleicher and Schuell, Kassel, Germany). Membranes were blocked for 2 h at room temperature in 140 mM NaCl, 3 mM KCl, 25 mM Tris-HCl (pH 7.4), 0.1% Tween 20 (tris-buffered saline Tween-20), 5% non-fat milk, and then immunoblotted with monoclonal mouse anti-DEPDC1 antibody (Abnova, Taiwan, China), rabbit anti-p27^Kip1^, anti-p53, anti-Phospho-p53 (Ser-15 or Ser-20), and mouse anti-p21^Cip1^ antibodies (Cell Signaling Technology, Beverly, MA, USA). As a control for protein loading, we used a mouse monoclonal anti-β-actin antibody (Sigma, St Louis, MO, USA). The primary antibodies were visualized with goat anti-rabbit (Sigma) or goat anti-mouse (Bio-Rad, Hercules, CA, USA) peroxidase-conjugated antibodies by an enhanced chemiluminescence detection system. Blots were quantified by densitometry using NIH ImageJ software (National Institutes of Health, Bethesda, MD, USA) and protein expression normalized according to β-actin expression.

### Obtaining of HMCLs Conditionally Expressing *shDEPDC1A*

Three human multiple myeloma cell lines (HMCLs) were used including two wild type *TP53* HMCLs (XG7 and XG19) and one *TP53*-mutated HMCL (XG2). HMCLs (XG2, XG7 and XG19) were first transduced with a lentivirus containing the tetracycline repressor (TR) that repressed Tet operator (Tet)O (HMCL-TR).

HMCL-TRs were then transduced with a lentiviral vector containing a *DEPDC1A* shRNA (shD1) linked with *GFP* gene under the control of Tet operator (HMCL-TR-shD1). The *DEPDC1A* shRNA targets the coding sequence of *DEPDC1A* and was purchased from Invitrogen (Invitrogen, Carlsbad, USA). In some experiments, HMCL-TR-shD1 were also cotransduced with a lentivirus containing *DEPDC1A* gene under the control of Tet operator (HMCL-TR-DEP-shD1). The lentivirus expressing *shDEPDC1A* or *DEPDC1A* were produced using Invitrogen methodology(Invitrogen, Carlsbad, USA). The human 293FT cell line was cultured in Dulbecco’s modified Eagle’s medium and supplemented with 10% defined fetal bovine serum, 500 µg/ml geneticin, 4 mM *L*-glutamine, and 1 mM MEM sodium pyruvate. The day before transfection, cells were plated into a 10 cm tissue culture plate to 90%–95% confluence. Nine µg of ViraPower packaging mix, 9 µg of pLenti3.3TR plasmid, and 9 µg of pLenti4 plasmid containing or not *shDEPDC1A* (Invitrogen, Carlsbad, USA). were co-transfected into 293FT cells using 36 µl Lipofectamine 2000 reagent. The nucleotide sequence of shDEPDC1A used was: 5′-TGCTGTTAAGTGGCGAAGTTGCAGGAGTTTTGGCCACTGACTGACTCCTGCAATCGCCACTTAA-3′. To produce the *DEPDC1A* lentivirus, 9 mg of pLenti6.3 plasmid (Invitrogen) containing or not *DEPDC1A* cDNA (comprising the coding sequence only, purchased from ImaGenes (ImaGenes GmbH, Berlin, Germany) were used. After forty-eight hours, culture supernatant was collected, concentrated, and viral titer determined. The HMCLs expressing *TR* transgene (HMCL-TR) or *TR* and *shDEPDC1A* (HMCL-TR-shDEPDC1A) or *DEPDC1A* were obtained applying selection antibiotics. To exclude off-targets of *DEPDC1A* shRNA, we used a siRNA targeting the non-coding 3′ part of *DEPDC1A* mRNA (position 835 in the sequence) and the control scrambled siRNA, purchased from Invitrogen (Invitrogen, Carlsbad, USA). To introduce the siRNA into cells, XG7 cells were transfected with lipofectamine RNAiMAX (5 µl, Invitrogen, Carlsbad, USA) and 50 nM siRNA in RPMI 1640 culture medium and 10% fetal bovine serum. Transfected cells are cultured with RPMI 1640 medium, 10% fetal bovine serum and 2 ng/ml IL6.

Gene expression in *shDEPDC1A* shRNA-expressing cells was evaluated performing three independent experiments (control cells and cells expressing the shRNA) for each myeloma cell line treated for 5 days (XG7 and XG19 HMCLs) or 3 days (XG2 cells) with dox and hybridizing six microarrays (Affymetrix U133 plus 2.0) per cell line (3 controls and 3 *shDEPDC1A*).

### Cell Growth, Cell Cycle, Apoptosis and Immunophenotypic Analysis

HMCLs were cultured for 4 days in 96-well flat-bottom microtiter plates in complete culture medium with IL-6 and cell growth was evaluated by quantifying intracellular ATP amount with a Cell Titer Glo Luminescent Assay (Promega, Madison, WI) with a Centro LB 960 luminometer (Berthold Technologies, Bad Wildbad, Germany). In some experiments, doxycycline (1 µg/ml) was added to induce *shDEPDC1A* expression. The cell cycle was assessed using 4′,6-diamidino-2-phenylindole (DAPI) staining (Sigma Aldrich) and ModFit analysis (Verity Software House, Topsham, ME). Cells in the S phase were quantified using incubation with bromodeoxyuridine (BrdU) for 1 hour, and labelling with an anti-BrdU antibody (APC BrdU flow kit, BD Pharmingen, Le Pont De Claix, France) as indicated [43]. Apoptotic cells were detected using phycoerythrin-conjugated annexin V (PE-annexin V, BD Pharmingen). For the immunophenotypic analysis, cells were stained using an anti-CXCR4 mAb, an anti-IL6R mAb and an anti-CD38 mAb (all from BD Biosciences).

### Statistical Analysis

Gene expression data were normalized with the MAS5 algorithm and analyzed with our bioinformatics platform - RAGE, http://rage.montp.inserm.fr/ [44] and Amazonia, http://amazonia.montp.inserm.fr/) [45] - and the SAM (Significance Analysis of Microarrays) software (www-stat.stanford.edu/∼tibs/SAM/). Statistical comparisons were done with Mann-Whitney, Chi-square, or unpaired or paired Student’s t-tests. The change in gene expression induced by *shDEPDC1A* in HMCLs was evaluated with Affymetrix GCOS (GeneChip Operating Software) software using Wilcoxon test and crossing, for each cell line, the gene lists of the 3 independent experiments. The advantage of Affymetrix GCOS software is to use the information provided by the 11 perfect-match (PM) and the 11 mismatch (MM) probes designed to build a given probe set signal, and avoids losing most of information given we have only 3 replicates and stringent multiple testing correction. Briefly, The analysis compares the difference values (PM-MM) of each probe pair in the baseline array to its matching probe pair on the experiment array. For each probe set a change *p*-value and an associated fold change is generated using Wilcoxon test. The *p*-value is then adjusted using Benjamini-Hochberg multiple testing correction.

## Supporting Information

Figure S1 **A.** Correlation (r = 0.7, *P* = .005) between *DEPDC1A* gene expression assayed both by real time RT-PCR or Affymetrix microarrays (probe set 222958_s_at) in 16 HMCLs. **B.** *DEPDC1A* expression in primary MMCs of the eight molecular groups of previously-untreated patients defined by UAMS. *DEPDC1A expression was significantly (*P* ≤.05) increased in patients in the proliferation (PR) group and decreased in patients with bone disease (LB), hyperdiploid (HY) or myeloid (MY) groups.
(TIF)

Figure S2 **Knockdown of** ***DEPDC1A*** **expression using inducible shRNA.** XG19-TR-shD1 cells were treated for 6 days with or not doxycycline (dox). Data shows the induction of GFP expression by dox treatment assayed using flow cytometry.
(TIF)

Figure S3 **Kinetics of** ***DEPDC1A*** **gene expression after doxycycline treatment.** XG19-TR-shD1 cells were treated for various days with or without doxycycline (dox) and *DEPDC1A* gene expression assayed using real time PCR. Results are the mean percentages of *DEPDC1A* gene expression in dox-treated cells compared to dox-untreated cells in 3 separate experiments. *indicates the mean percentage in the dox-treated cells is significantly different than that in the dox-untreated cells using a paired t test (*P*<.05).
(TIF)

Figure S4 **Overexpression of** ***DEPDC1A*** **abrogated the growth delay induced by a** ***DEPDC1A*** **siRNA.** *DEPDC1A* gene was overexpressed 8 fold in XG7 cells by lentiviral delivery of *DEPDC1A* coding sequence. *DEPDC1A* RNA was quantified by real time RT-PCR and results are mean values of 3 representative experiments. A. The siRNA targeting the non-coding part of *DEPDC1A* mRNA decreased by 47% the expression of endogenous *DEPDC1A* gene, and did not affect that of *DEPDC1A* transgene lacking the 3′ non-coding sequence. Results are mean values of real-time RT-PCR of 3 independent experiments. B. The siRNA targeting the non-coding part of *DEPDC1A* mRNA delayed the growth of XG7 cells but did not affect the growth of XG7 cells transduced with *DEPDC1A* transgene. The cell lines were cultured for 3 days after siRNA transfection. Data are the mean counts ± SD of viable cells using trypan blue exclusion of 3 separate experiments. D. The siRNA targeting the non-coding part of *DEPDC1A* mRNA delayed cell cycle in the G2 phase in XG7 cells, but did not affect cell cycle in XG7 cells transduced with *DEPDC1A* transgene lacking the 3′ non-coding sequence. Results are FACS cell cycle data of a representative experiment and the table below shows the mean values ± SD of cell cycle data of 3 experiments. *indicates the mean percentage is significantly different than that in cells treated with the scrambled siRNA using a paired t test (*P*<.05).
(TIF)

Figure S5 ***DEPDC1A*** **knockdown increases expression of markers of mature plasma cells in myeloma cell lines.** XG7-TR-shD1 cells were cultured with or without doxycycline (dox) for 6 days and cells were stained with phycoerythrin-conjugated anti-CXCR4 mAb, anti-IL6R mAb, anti-CD38 mAb (continuous line) or isotype-matched control mAbs (black full histogram). Facs data are those of one experiment representative of three. ΔMFI is the difference between the mean fluorescence intensity (MFI) with the antigen-specific antibody and MFI with isotype-matched control mAb. The table indicates the mean ratio of ΔMFI between dox-treated and dox-untreated XG19 myeloma cells in 3 independent experiments. *indicates a significant increase in CXCR4, IL6R or CD38 expression using a paired t-test (*P*<.05).
(TIF)

Table S1 **Clinical characteristics of patients of HM cohort.** Data are median values and ranges for age, serum monoclonal protein, serum-β-microglobulin and the Salmon-Durie and International Staging System (ISS) stages. NA, not available.
(PDF)

Table S2 **Cox univariate and multivariate analysis of overall survival in UAMS-TT2 patients’ cohorts.** The prognostic factors were tested as single variable or multi variables using a Cox-model. Hazard ratios (HR) and P-values are shown. NS, Not Significant at a 5% threshold; GPI, gene expression based proliferation index; PR, Proliferation stratification according to UAMS.
(PDF)

Table S3 **Genes deregulated in wild-type** ***TP53*** **XG7 human myeloma cell line.**
(XLS)

Table S4 **Genes deregulated in wild-type** ***TP53*** **XG19 human myeloma cell line.**
(XLS)

Table S5 **Genes deregulated in mutated** ***TP53*** **XG2 human myeloma cell line.**
(XLS)

Table S6 **Genes overexpressed in DEPDC1A high patients compared to DEPDC1 low patients.**
(XLS)
