# Supplementary figures and images for: Inhibition of DEPDC1A, a Bad Prognostic Marker in Multiple Myeloma, Delays Growth and Induces Mature Plasma Cell Markers in Malignant Plasma Cells
Source: PLoS One. 2013 Apr 30;8(4):e62752. doi: 10.1371/journal.pone.0062752 (PMC3640027; doi:10.1371/journal.pone.0062752)

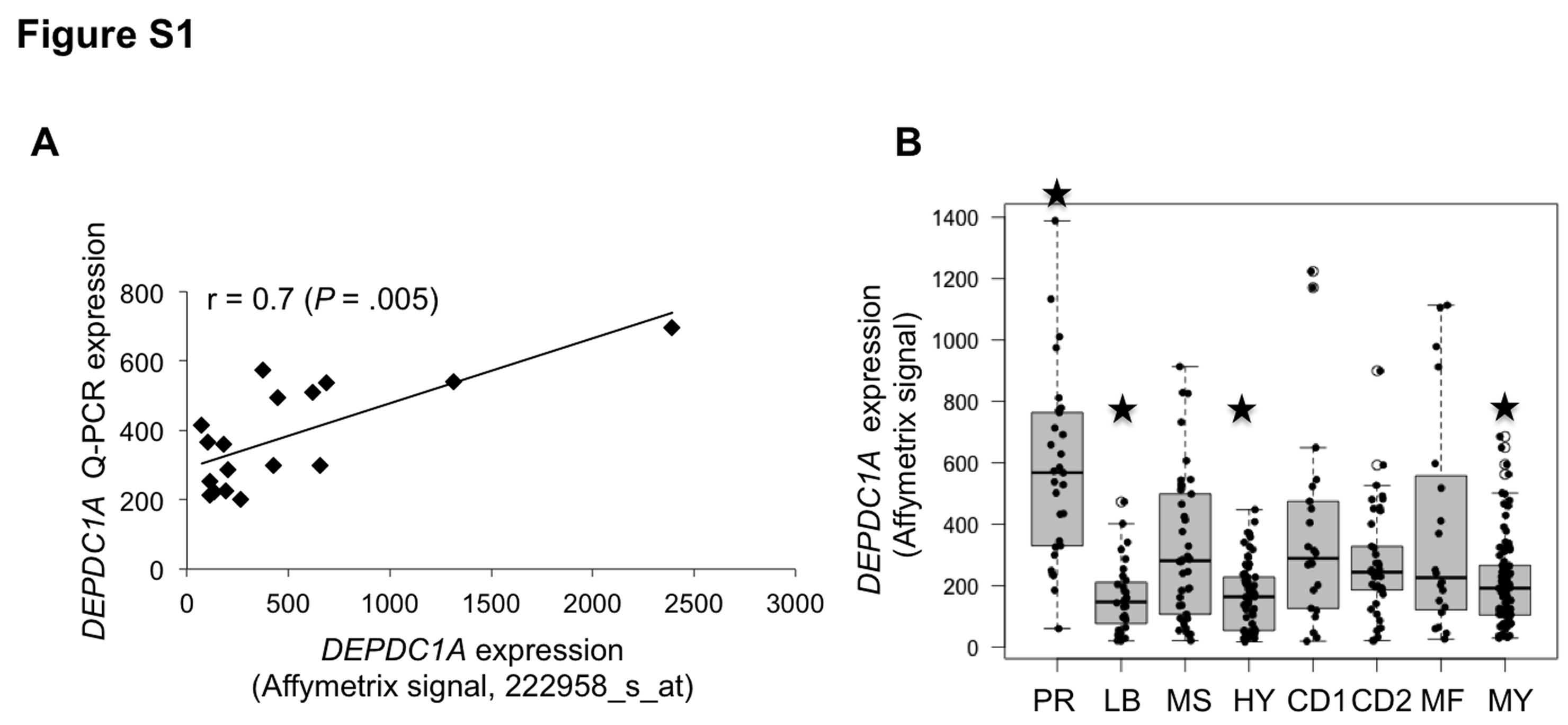

Supplement: Figure S1 — A. Correlation (r = 0.7, P = .005) between DEPDC1A gene expression assayed both by real time RT-PCR or Affymetrix microarrays (probe set 222958_s_at) in 16 HMCLs. B. DEPDC1A expression in primary MMCs of the eight molecular groups of previously-untreated patients defined by UAMS. *DEPDC1A expression was significantly (P ≤.05) increased in patients in the proliferation (PR) group and decreased in patients with bone disease (LB), hyperdiploid (HY) or myeloid (MY) groups. (TIF) [file pone.0062752.s001.tif]

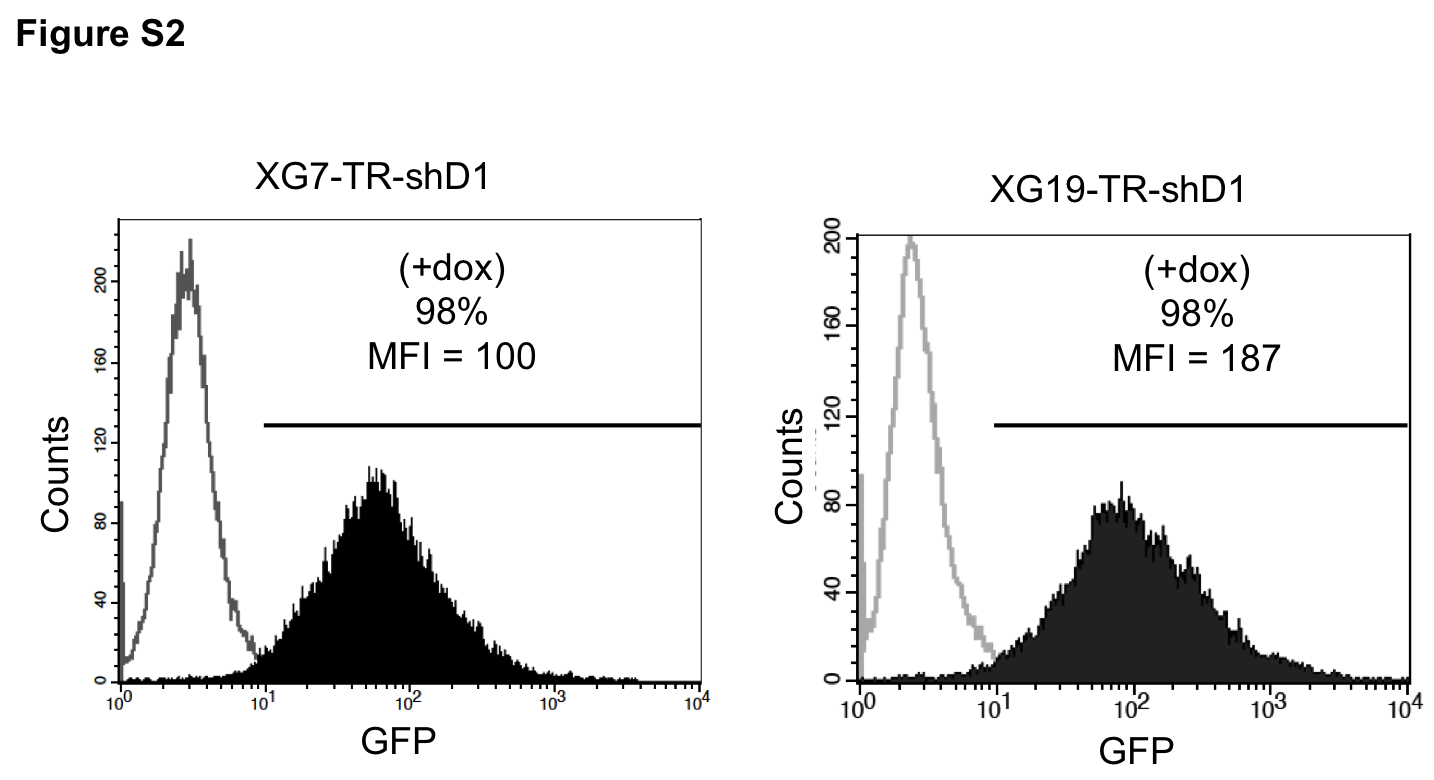

Supplement: Figure S2 — Knockdown of DEPDC1A expression using inducible shRNA. XG19-TR-shD1 cells were treated for 6 days with or not doxycycline (dox). Data shows the induction of GFP expression by dox treatment assayed using flow cytometry. (TIF) [file pone.0062752.s002.tif]

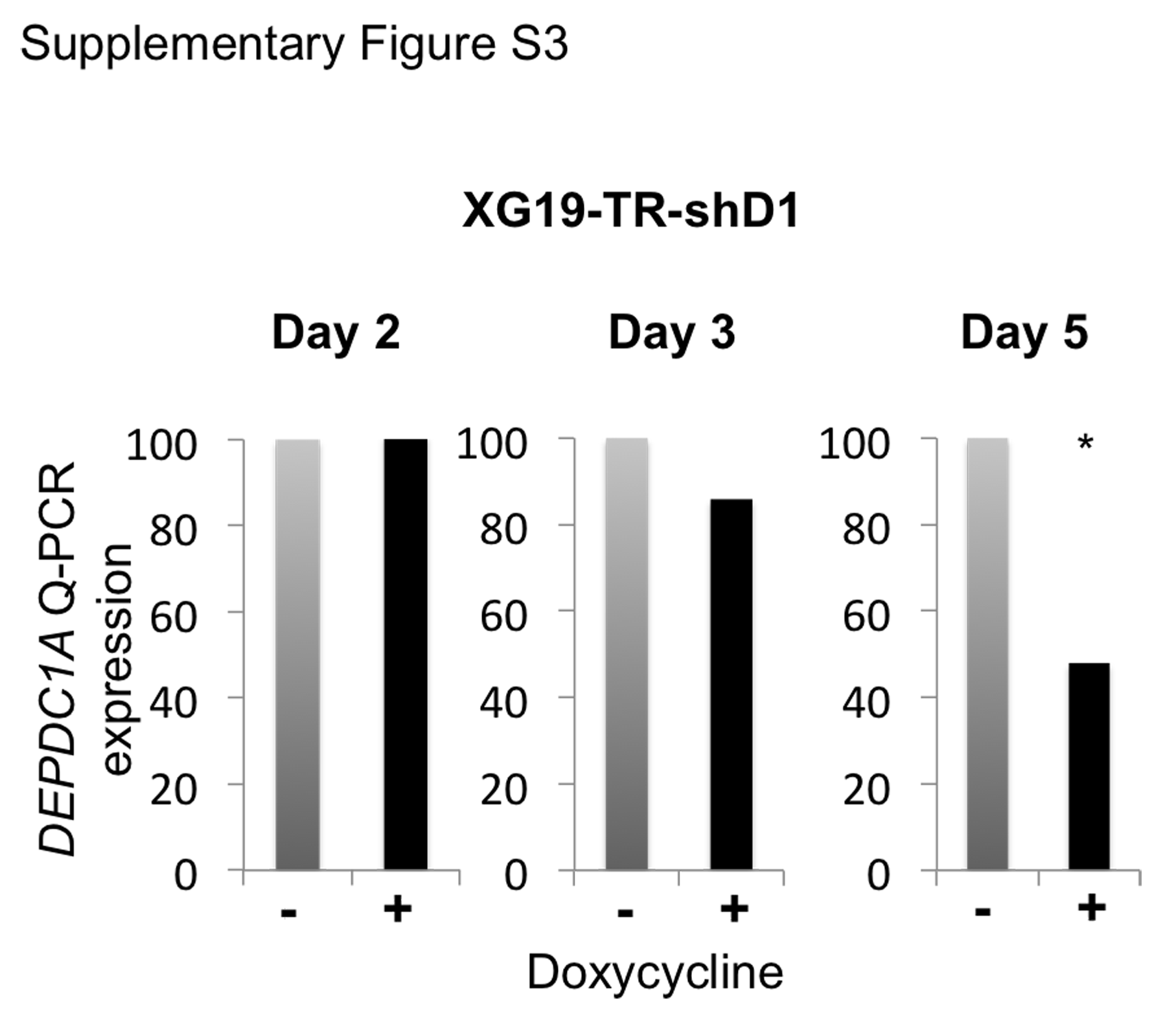

Supplement: Figure S3 — Kinetics of DEPDC1A gene expression after doxycycline treatment. XG19-TR-shD1 cells were treated for various days with or without doxycycline (dox) and DEPDC1A gene expression assayed using real time PCR. Results are the mean percentages of DEPDC1A gene expression in dox-treated cells compared to dox-untreated cells in 3 separate experiments. *indicates the mean percentage in the dox-treated cells is significantly different than that in the dox-untreated cells using a paired t test (P<.05). (TIF) [file pone.0062752.s003.tif]

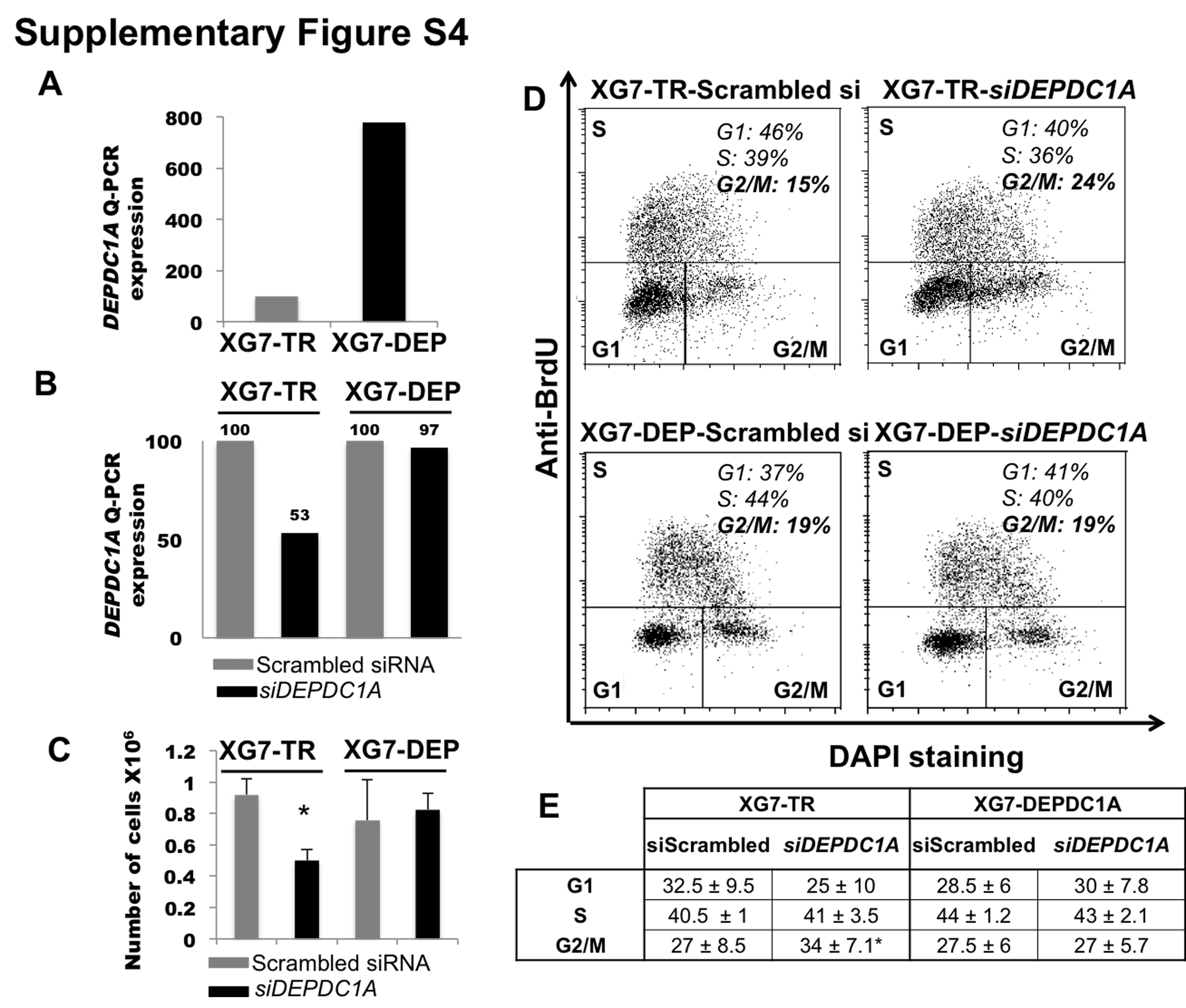

Supplement: Figure S4 — Overexpression of DEPDC1A abrogated the growth delay induced by a DEPDC1A siRNA. DEPDC1A gene was overexpressed 8 fold in XG7 cells by lentiviral delivery of DEPDC1A coding sequence. DEPDC1A RNA was quantified by real time RT-PCR and results are mean values of 3 representative experiments. A. The siRNA targeting the non-coding part of DEPDC1A mRNA decreased by 47% the expression of endogenous DEPDC1A gene, and did not affect that of DEPDC1A transgene lacking the 3′ non-coding sequence. Results are mean values of real-time RT-PCR of 3 independent experiments. B. The siRNA targeting the non-coding part of DEPDC1A mRNA delayed the growth of XG7 cells but did not affect the growth of XG7 cells transduced with DEPDC1A transgene. The cell lines were cultured for 3 days after siRNA transfection. Data are the mean counts ± SD of viable cells using trypan blue exclusion of 3 separate experiments. D. The siRNA targeting the non-coding part of DEPDC1A mRNA delayed cell cycle in the G2 phase in XG7 cells, but did not affect cell cycle in XG7 cells transduced with DEPDC1A transgene lacking the 3′ non-coding sequence. Results are FACS cell cycle data of a representative experiment and the table below shows the mean values ± SD of cell cycle data of 3 experiments. *indicates the mean percentage is significantly different than that in cells treated with the scrambled siRNA using a paired t test (P<.05). (TIF) [file pone.0062752.s004.tif]

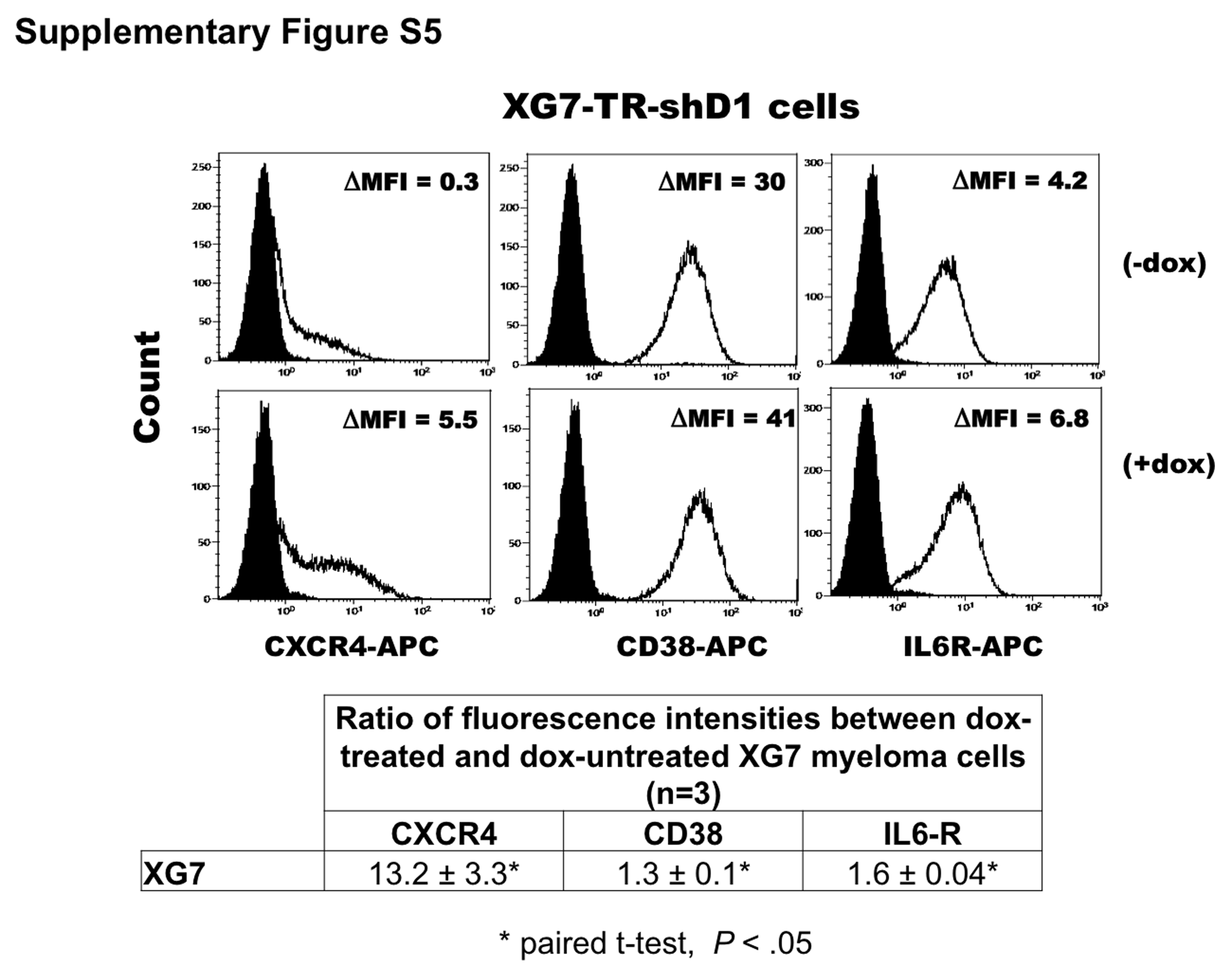

Supplement: Figure S5 — DEPDC1A knockdown increases expression of markers of mature plasma cells in myeloma cell lines. XG7-TR-shD1 cells were cultured with or without doxycycline (dox) for 6 days and cells were stained with phycoerythrin-conjugated anti-CXCR4 mAb, anti-IL6R mAb, anti-CD38 mAb (continuous line) or isotype-matched control mAbs (black full histogram). Facs data are those of one experiment representative of three. ΔMFI is the difference between the mean fluorescence intensity (MFI) with the antigen-specific antibody and MFI with isotype-matched control mAb. The table indicates the mean ratio of ΔMFI between dox-treated and dox-untreated XG19 myeloma cells in 3 independent experiments. *indicates a significant increase in CXCR4, IL6R or CD38 expression using a paired t-test (P<.05). (TIF) [file pone.0062752.s005.tif]
