# Supplementary material for: Inhibition of DEPDC1A, a Bad Prognostic Marker in Multiple Myeloma, Delays Growth and Induces Mature Plasma Cell Markers in Malignant Plasma Cells
Source: PLoS One. 2013 Apr 30;8(4):e62752. doi: 10.1371/journal.pone.0062752 (PMC3640027; doi:10.1371/journal.pone.0062752)
Supplement: Table S1 — Clinical characteristics of patients of HM cohort. Data are median values and ranges for age, serum monoclonal protein, serum-β-microglobulin and the Salmon-Durie and International Staging System (ISS) stages. NA, not available. (PDF) [file pone.0062752.s006.pdf]

Table S1. Clinical patient data.

	Characteristic	HM (n=206)
Age (median[range])		57[27-73]
Monoclonal protein		
	IgG	120
	IgA	46
	Bence Jones	35
	Asecretory	4
	IgD	1
	NA	0
Myeloma in Durie and Salmon (SD) sage		
	I	22
	II	31
	III	153
Myeloma in ISS stage		
	I	97
	II	73
	III	33
	NA	3
Serum β2-microglobulin (median[range])		2.9[1.3-53.6]
