# Supplementary material for: Inhibition of DEPDC1A, a Bad Prognostic Marker in Multiple Myeloma, Delays Growth and Induces Mature Plasma Cell Markers in Malignant Plasma Cells
Source: PLoS One. 2013 Apr 30;8(4):e62752. doi: 10.1371/journal.pone.0062752 (PMC3640027; doi:10.1371/journal.pone.0062752)
Supplement: Table S2 — Cox univariate and multivariate analysis of overall survival in UAMS-TT2 patients’ cohorts. The prognostic factors were tested as single variable or multi variables using a Cox-model. Hazard ratios (HR) and P-values are shown. NS, Not Significant at a 5% threshold; GPI, gene expression based proliferation index; PR, Proliferation stratification according to UAMS. (PDF) [file pone.0062752.s007.pdf]

	**Univariate Cox analysis - Overall survival**	
	**LR-TT2**	
	HR	*P*
DEPDC1A	2.3	2.10E-05
GPI	1.8	2.20E-04
PR stratification	2.6	1.70E-04

	**Multivariate Cox analysis - Overall survival**	
	**LR-TT2**	
	HR	*P*
DEPDC1A	1.8	1.40E-02
GPI	1.4	NS
DEPDC1A	2	1.60E-03
PR stratification	1.8	0.03

	**Multivariate Cox analysis - Overall survival**	
	**LR-TT2**	
	HR	*P*
DEPDC1A	1.7	0.028
GPI	1.2	NS
PR stratification	1.6	NS

Table S2. Cox univariate and multivariate analysis of overall survival in UAMS-TT2 patients' cohorts.
